# Glypican 6 is a putative biomarker for metastatic progression of cutaneous melanoma

**DOI:** 10.1371/journal.pone.0218067

**Published:** 2019-06-14

**Authors:** Yuanyuan Li, Melissa Li, Igor Shats, Juno M. Krahn, Gordon P. Flake, David M. Umbach, Xiaoling Li, Leping Li

**Affiliations:** 1 Biostatistics and Computational Biology Branch, National Institute of Environmental Health Sciences, Durham, North Carolina, United States of America; 2 Laboratory of Signal Transduction, National Institute of Environmental Health Sciences, Durham, North Carolina, United States of America; 3 Genome Integrity & Structural Biology Laboratory, National Institute of Environmental Health Sciences, Durham, North Carolina, United States of America; 4 Cellular and Molecular Pathology Branch, National Institute of Environmental Health Sciences, Durham, North Carolina, United States of America; University of South Alabama Mitchell Cancer Institute, UNITED STATES

## Abstract

Due to the poor prognosis of advanced metastatic melanoma, it is crucial to find early biomarkers that help identify which melanomas will metastasize. By comparing the gene expression data from primary and cutaneous melanoma samples from The Cancer Genome Atlas (TCGA), we identified GPC6 among a set of genes whose expression levels can distinguish between primary melanoma and regional cutaneous/subcutaneous metastases. Glypicans are thought to play a role in tumor growth by regulating the signaling pathways of Wnt, Hedgehogs, fibroblast growth factors (FGFs), and bone morphogenetic proteins (BMPs). We showed that GPC6 expression was up-regulated in a melanoma cell line compared to normal melanocytes and in metastatic melanoma compared to primary melanoma. Furthermore, GPC6 expression was positively correlated with genes largely involved in cell adhesion and migration in both melanoma samples and in RNA-seq samples from other TCGA tumors. Our results suggest that GPC6 may play a role in tumor metastatic progression. In TCGA melanoma samples, we also showed that GPC6 expression was negatively correlated with miR-509-3p, which has previously been shown to function as a tumor suppressor in various cancer cell lines. We overexpressed miR-509-3p in A375 melanoma cells and showed that GPC6 expression was significantly suppressed. This result suggested that GPC6 was a putative target of miR-509-3p in melanoma. Together, our findings identified GPC6 as an early biomarker for melanoma metastatic progression, one that can be regulated by miR-509-3p.

## Introduction

The Cancer Genome Atlas (TCGA) project has generated a large amount of data using several platforms, including RNA-seq, applied to the same tissue specimens for a variety of tumors like melanoma [1]. These data provide unprecedented information about the molecular map of tumors. Previously, using TCGA data, we showed that primary melanomas are heterogeneous at the gene expression level, and that the degree of loss of epithelium-characteristic gene expression in those melanomas is correlated with predicted metastatic progression [2].

Despite its relatively low incidence rate, skin cutaneous melanoma (SKCM) is the deadliest type of skin cancer due to its invasiveness. For patients with stage IV melanoma with a metastasis spread to the lymph nodes or other organs, the median survival is 8–9 months [3]. The development of BRAF inhibitors, such as vemurafenib and dabrafenib [4], have dramatically altered the paradigm of melanoma treatment and improved patient survival. Unfortunately, many patients eventually develop resistance to these drugs. To address this challenge, new targeted drugs are being developed and immunotherapy has shown great promise (for a recent review, see [5]).

Although it can be deadly, melanoma is often curable when diagnosed early. Thus, early detection and intervention can be lifesaving and markers for melanoma progression would be informative. Herein, we analyzed the RNA-seq gene expression data from TCGA for 74 primary melanomas and 66 melanomas that had metastasized to regional cutaneous/subcutaneous tissues (including satellite and in-transit metastases). We aimed to identify genes whose expression levels distinguish primary melanoma from cutaneous/subcutaneous melanoma, hoping to identify biomarkers that are indicative of early melanoma metastatic progression. In this report, we focused on a new putative melanoma gene, glypican 6 (GPC6).

Glypicans are a family of heparan sulfate proteoglycans that are linked to the extracellular cell surface of the plasma membrane. They are thought to regulate the signaling of Wnt, Hedgehogs, fibroblast growth factors (FGFs), and bone morphogenetic proteins (BMPs) [6–12]. Six glypicans have been identified in mammals (GPC1 to GPC6) and two in Drosophila, where they play important roles in development (reviewed in [11–13]). GPC6 is the newest member of the family, is most homologous to GPC4, and is ubiquitously expressed [14].

GPC6 has also been implicated in many cancers and other diseases. Exome sequencing identified GPC6 among genes that were recurrently mutated across individual prostate tumors from different patients [15]. In a large-scale genome-wide association study (GWAS), Amankwah et al. identified a single-nucleotide polymorphism (SNP) (rs17702471) in GPC6/GPC5 that was associated with an increased risk of invasive epithelial ovarian carcinoma [16]. Loftus et al. [17] demonstrated that HIF1α signaling and hypoxia in melanocytes directly regulate the expression of GPC6 and that increased expression of GPC6 was positively associated with poor survival in melanoma. GPC6 was among a group of genes whose expression levels were significantly correlated with reduced time of disease-free status in melanoma [17]. Taken together, these studies demonstrated that GPC6 functions in tumor progression.

Using RNA-seq gene expression data from TCGA, we identified GPC6 among a set of genes whose expression levels can distinguish primary skin melanoma from melanoma metastasized to cutaneous/subcutaneous tissue. In this report, we used both computational and experimental approaches to try to understand the role of GPC6 in the progression of melanoma.

## Materials and methods

### Data

The Cancer Genome Atlas (TCGA) collected melanoma tissue samples from one of the four tissue sites: skin (primary tumor), and metastases to regional cutaneous/subcutaneous tissues, lymph nodes, or more distant sites (the latter three referred to collectively as metastatic tumors). For simplicity, we refer to the four categories of melanoma samples simply as: primary, cutaneous, lymph, and distant metastases with 74, 66, 195, and 54 RNA-seq samples (389 total), respectively. We downloaded (May 2015) UNC RNASeqV2 and SKCM level 3 expression data from the TCGA data portal (https://portal.gdc.cancer.gov/). We log_2_-transformed the normalized read counts (per million reads mapped) for RNA-seq data (all values less than 1 were assigned value 1 before transformation), but we carried out no further normalization. We also downloaded RNA-seq data for 1,156 cancer cell lines from the Cancer Cell Line Encyclopedia (CCLE) (https://portals.broadinstitute.org/ccle).

### Computational method

In preliminary work, we concluded that it is not feasible to identify a set of genes whose expression levels can correctly classify all four classes of melanoma samples (data not shown). Thus, we decided to focus on distinguishing primary melanoma (74 samples) from cutaneous/subcutaneous (66 samples) melanoma. We randomly divided the data into a training (75% of the samples) and a testing set (25% of the samples). We used the training set to train the classification algorithm (GA/KNN) [18, 19] and evaluated training performance through a leave-one-out cross-validation procedure. The prediction accuracies may vary depending on which samples are assigned to the training and testing sets. To avoid idiosyncrasies from use of a single random assignment, we carried out 100 independent random training and testing assignments and obtained training-set and testing-set prediction accuracies from each. On average, each sample was placed 75 times in the training set and 25 times in the testing set and predicted the corresponding number of times. We used the average prediction accuracies from the ~75 training predictions and from the ~25 testing predictions for each sample as the overall prediction accuracies for that sample in training and testing sets, respectively. In our analysis, for a given training/testing partition, we collected 5,000 near-optimal feature sets, resulting in 5,000 classifications of the training- and testing-set samples.

The details of the GA/KNN algorithm can be found in [18, 19]. The chromosome length was set to 20 (a 20-gene set) and the population size was set to 300. The maximal number of generations of “genetic evolution” was set to be 1000. For the k-nearest neighbor (KNN) classification, k was set to 5 with a majority “voting” rule.

To identify putative miR-509-3p binding sites in the 3’-untranslated region (UTR) of human GPC6 gene, we downloaded the 3’-UTR sequence of human GPC6 (NM_005708) from the UCSC genome browser (build hg38) (https://genome.ucsc.edu/). We used a custom C code to search for segments in the GPC6 sequence that are complementary to the 3’- to 5’- seed sequence (seed length = 8) of human miR-509-3p. A putative target site was declared when seven or more of the eight nucleotides were complementary (one G:U pairing was allowed).

### Experimental method

A375 melanoma cells (150,000)/well were reverse transfected in 6-well plates with 4nM negative control mimic (catalog # 4464077, Thermo Fisher) or miR-509-3p mimic (assay ID MC12984, Thermo Fisher) with Lipofectamine RNAiMax reagent (catalog # 13778030, Thermo Fisher) according to the manufacturer’s protocol. After 48 hours of transfection, cells were collected in TRIzol (catalog # 15596018, Thermo Fisher) and total RNA was extracted according to the manufacturer’s protocol. cDNA was synthesized with the High-Capacity cDNA Reverse Transcription Kit (catalog # 4368814, Thermo Fisher) according to the manufacturer’s protocol. qPCR was performed using iQ SYBR Green Supermix (catalog # 1708880, Bio-Rad) on a CFX96 Touch Real-Time PCR Detection System (Bio-Rad) with Taqman Assays for miR-509-3p (assay ID: 002236, Thermo Fisher), GPC6 (assay ID: Hs00170677_m1, Thermo Fisher), and Rplp0 (assay ID: HS99999902_m1, Thermo Fisher). Relative quantitation of target transcript expression was calculated using the ddCT method using Rplp0 as the endogenous control.

## Results

### Classification accuracies

The mean and median prediction accuracies were 93.5% and 97.8% for primary and cutaneous samples in the training sets, and 77.5% and 83.0% for samples in the testing sets (Table 1). The prediction accuracies for the training set were higher than those for the testing set, suggesting some training bias existed. Nonetheless, 80% of the time, the gene signatures (sets of 20 genes) selected by the GA/KNN algorithm could correctly assign an RNA-seq sample as either a primary melanoma or a metastasis to a regional cutaneous/subcutaneous tissue.

**Table 1:** Summary statistics of classification performances.

Melanoma tissue site	Min.	1^st^ Quartile	Median	Mean	3^rd^ Quartile	Max.
	Training set performance					
primary	0.428	0.935	0.984	0.934	0.996	1.000
cutaneous	0.420	0.943	0.975	0.936	0.990	0.999
Overall	0.420	0.937	0.978	0.935	0.994	1.000
	Testing set performance					
primary	0.124	0.688	0.862	0.781	0.962	0.997
cutaneous	0.144	0.689	0.819	0.768	0.912	0.993
Overall	0.124	0.686	0.830	0.775	0.943	0.997

### Top genes whose expression levels distinguish between primary and cutaneous melanoma

The ten most frequently selected genes from all 100 training/testing partitions were FABP4, SFRP4, CILP, SLITRK4, EBF2, PRG4, KRT6B, GPC6, KRT17, and OGN. All genes except the two keratin genes (KRT6B and KRT17) were up-regulated in cutaneous melanoma compared to primary melanoma (S1 Fig). Gene ontology (GO) analysis [20] showed that the top 300 genes were significantly enriched in GO terms: signal transduction, cell communication, cell structure and motility, and others (Table 2). Some of the other notable genes include genes in cell adhesion (ADAMTS6, ADAMTS9, ADAMTS12, FAT2, FAT3, COL17A1, DSC2, LAMC2, LARRC15, NTN1, ODZ2, PVRL4, PCDH17, SEMA3G, SCUBE3, and SLIT2), epithelial-mesenchymal transition (EMT) (ANGPTL1 and HMGA2), Hedgehog signaling (BMP8B, DHH, WNT4, WNT5A, WNT7B, and WNT11), MAPK pathway (DUSP4, DUSP5, IL17D, and RAPGEF5), receptor tyrosine kinase pathway (FLT1, PDGFRL, SH2B2 and TIE1), transcription factors (GLIS1), and Wnt signaling pathway (APCDD1, WNT4, WNT5A, WNT7B, and WNT11).

**Table 2:** Significant GO terms for the top 300 genes that distinguish primary from cutaneous melanoma.

Biological process	Number of genes in GO term	Multiple-testing-adjusted p-value
Signal transduction	80	0.0040
Cell communication	38	0.0021
Developmental processes	56	0.0022
Cell structure and motility	33	0.013
Mesoderm development	21	0.011
Cell adhesion-mediated signaling	16	0.018
Other neuronal activity	9	0.029
Muscle development	9	0.029
Cell structure	21	0.045

**Fig 1:**
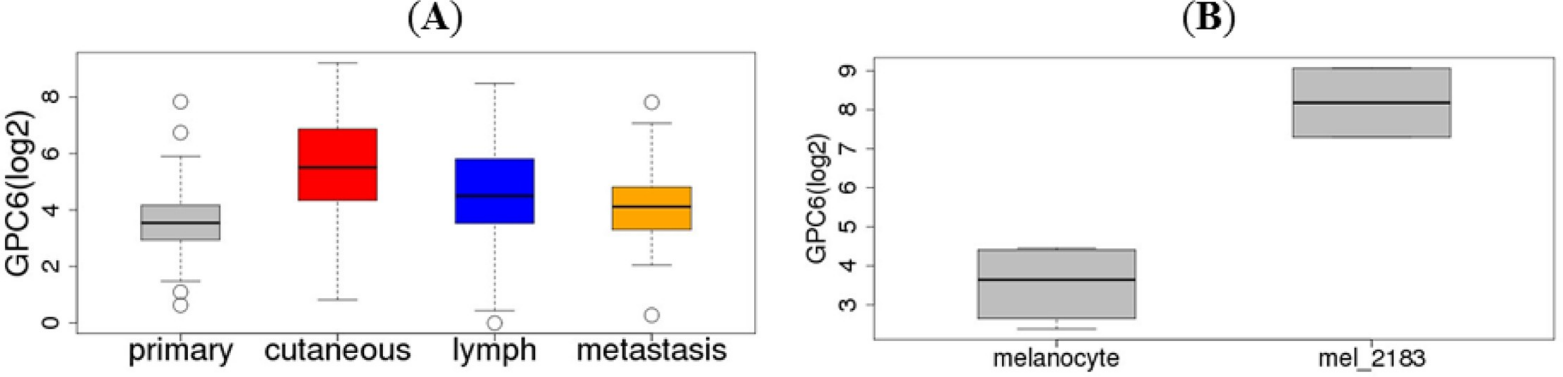
GPC6 expression in normal and melanoma cell lines and TCGA melanoma tumors. Data from the two platforms were not normalized for direct comparison. (A) GPC6 expression in TCGA melanoma samples from four tissue sites (RNA-seq from TCGA). (B) Boxplots of the expression of GPC6 in normal melanocytes (four replicates) and melanoma cell line (mel-2183, two replicates) measured by Affymetrix arrays (GSE15805).

Among the top ten genes whose expression levels distinguish primary from metastatic melanoma, we focused our subsequent analyses on a putative novel biomaker, GPC6, whose expression level was up-regulated in metastatic melanoma compared to primary melanoma (Fig 1A).

### Up-regulation of GPC6 expression in melanoma

Using public array gene expression data from the Encyclopedia of DNA Elements (ENCODE), we found that GPC6 was also up-regulated in a melanoma cell line (mel_2183) compared to normal melanocytes (GSE15805) (Fig 1B) and is overexpressed in metastatic melanoma compared to primary melanoma (Fig 1A). Next, we investigated a possible mechanism for GPC6 overexpression in melanoma.

### GPC6 is a putative target of miR-509-3p

**Fig 2:**
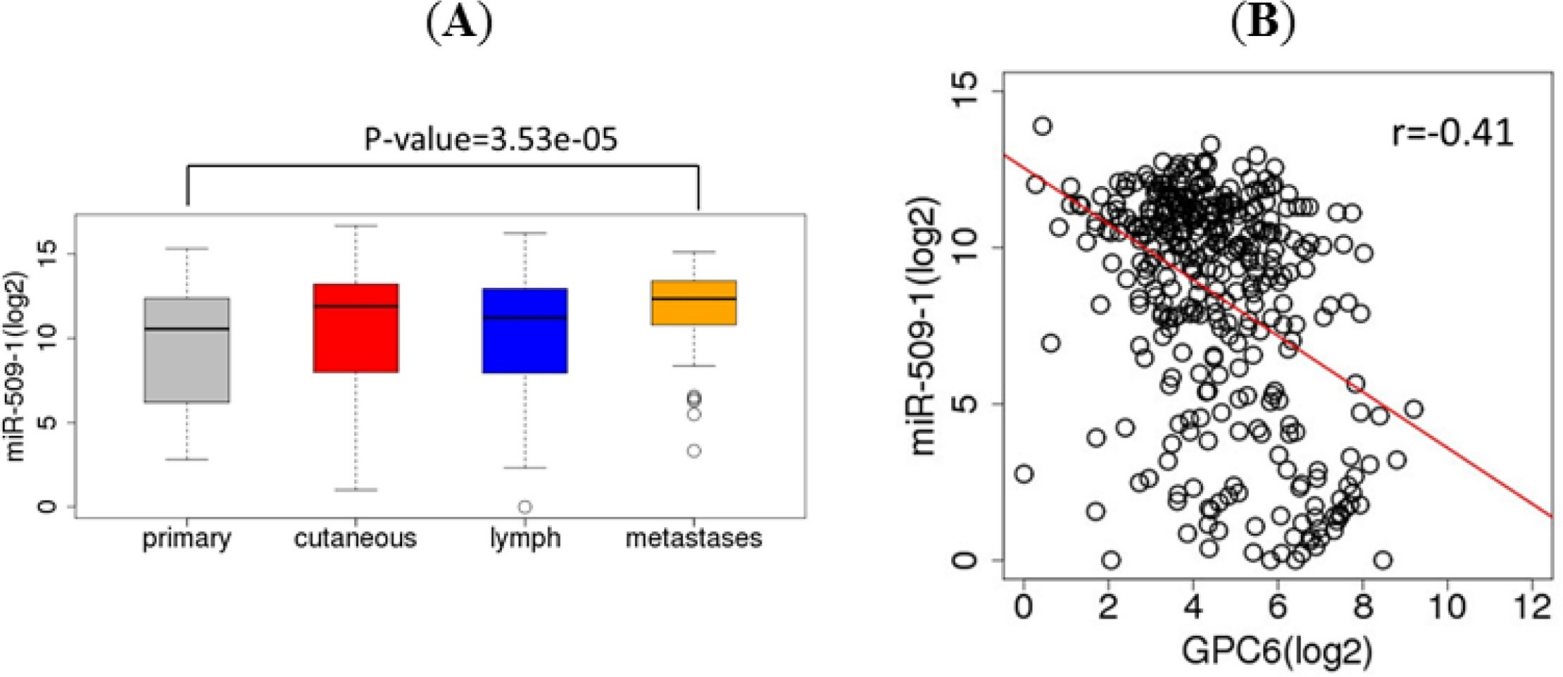
miR-509-1 expression and correlation with GPC6. (A) Boxplots of miR-509-1 expression levels in TCGA melanoma samples for the four tissue sites. (B) Scatter plot of miR-509-1 and GPC6 expression levels in TCGA melanoma samples (N = 389). The red line indicates the regression line.

**Fig 3:**
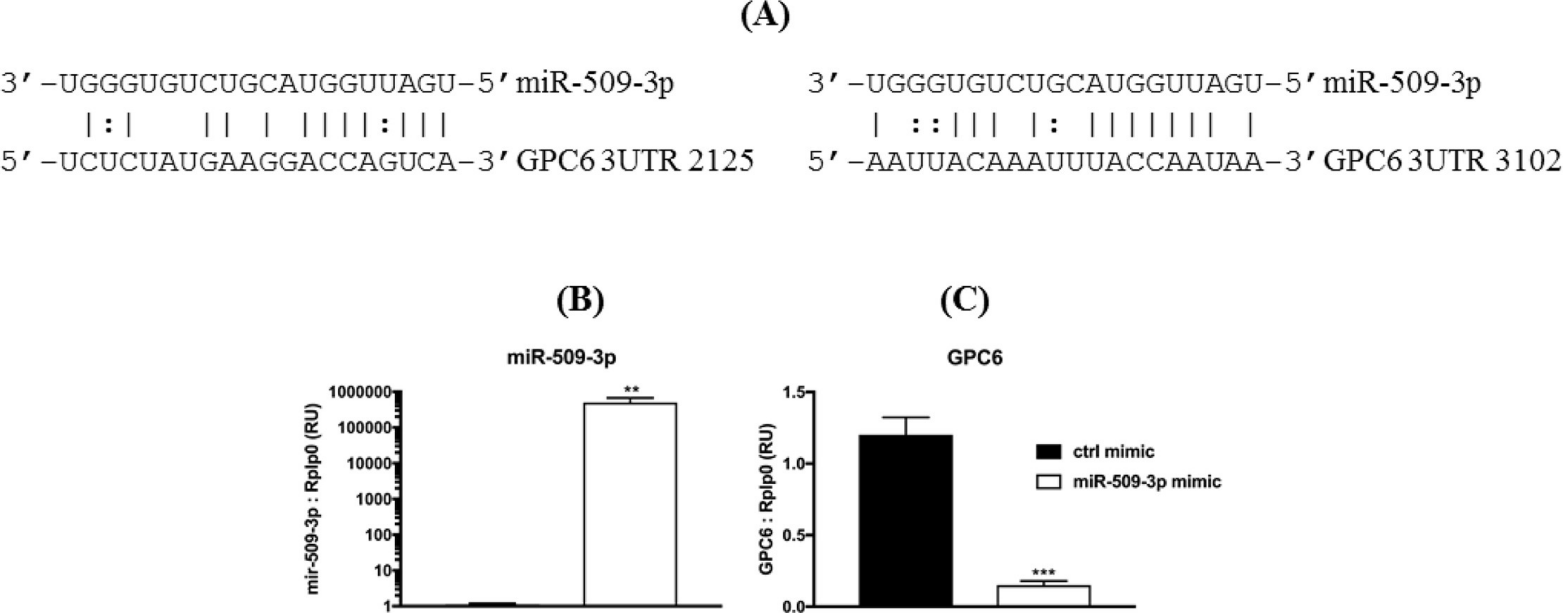
GPC6 is a putative target of miR-509-3p and overexpression of miR-509-3p downregulates GPC6 mRNA levels. (A) Predicted binding sites of miR-509-3p on the 3’-UTR of human GPC6 gene (NM005708). (B-C) A375 cells were transfected with 4nM negative control mimic (mimic ctrl) or miR-509-3p mimic for 48 hours. RNA was collected and RNA levels of miR-509-3p (B) and GPC6 (C) were measured using Taqman assays relative to Rplp0 from n = 3 experiments. Means ± S.D. **, p < 0.001; ***, p < 0.0001 for miR-509-3p mimic versus mimic control.

There are three microRNA-509 genes (miR-509-1, miR-509-2, and miR-509-3) in TCGA samples that all produce two mature forms of microRNAs (miR-509-5p and miR-509-3p). The expression levels of the three microRNAs were highly correlated (r = 0.997 − 0.999, Spearman correlation). Because of their high correlations, we used miR-509-1 as the representative of the three. The expression level of miR-509-1 in normal human melanocytes is not known. However, we found that miR-509-1 had higher expression level in melanoma distant metastases than in primary melanoma in TCGA samples (p = 3.53E-05, t test, two-sided) (Fig 2A). miR-509-1 expression was negatively correlated with that of GPC6 in TCGA melanoma samples (r = -0.41, Spearman correlation) (Fig 2B). Previously, Pan et al. [21] showed that GPC6 was among a few genes that were down-regulated in two ovarian cancer cell lines (OVCAR8 and HEYA8) transfected with miR-509-3p mimics relative to untreated controls. We identified two putative miR-509-3p binding sites in the 3’-UTR of human GPC6 gene (Fig 3A) and hypothesized that GPC6 is a putative target of miR-509-3a in melanoma cells. To test this hypothesis, we transfected A375 melanoma cells with negative control mimic or miR-509-3p mimic. After 48 hours, we collected total RNA and measured levels of miR-509-3p and GPC6. We found that miR-509-3p levels were significantly overexpressed with miR-509-3p mimic (Fig 3B), as expected. Furthermore, with miR-509-3p overexpression, GPC6 levels were significantly reduced (by 83–88%) (Fig 3C). GPC6 expression was up-regulated in TCGA metastatic melanoma samples compared to normal samples, however, its expression levels showed a metastasis stage-dependent decrease (Fig 1A) with an overall lowest expression in tumors metastasized to distant organs. Conversely miR-509-3p had the highest expression in distant metastases compared to primary melanoma tumors (Fig 2A). Those data are consistent with the notation that miR-509-3p may suppress GPC6 expression.

### Genes coordinately expressed with GPC6 are largely involved in cell adhesion

Analysis of all 389 melanoma samples revealed 82 genes whose expression levels were strongly positively correlated (r ≥ 0.5, Spearman correlation), and seven that were strongly negatively correlated (r ≤ -0.5, Spearman correlation) with GPC6. Among the 82 genes, 14 (EDIL3, CHD11, COL3A1, COL8A1, COL12A1, ECM2, LAMA2, NRP1, NTM, POSTN, PCDH17, ROR2, TNFAIP6, and VCAN) are known to be involved in cell adhesion.

**Fig 4:**
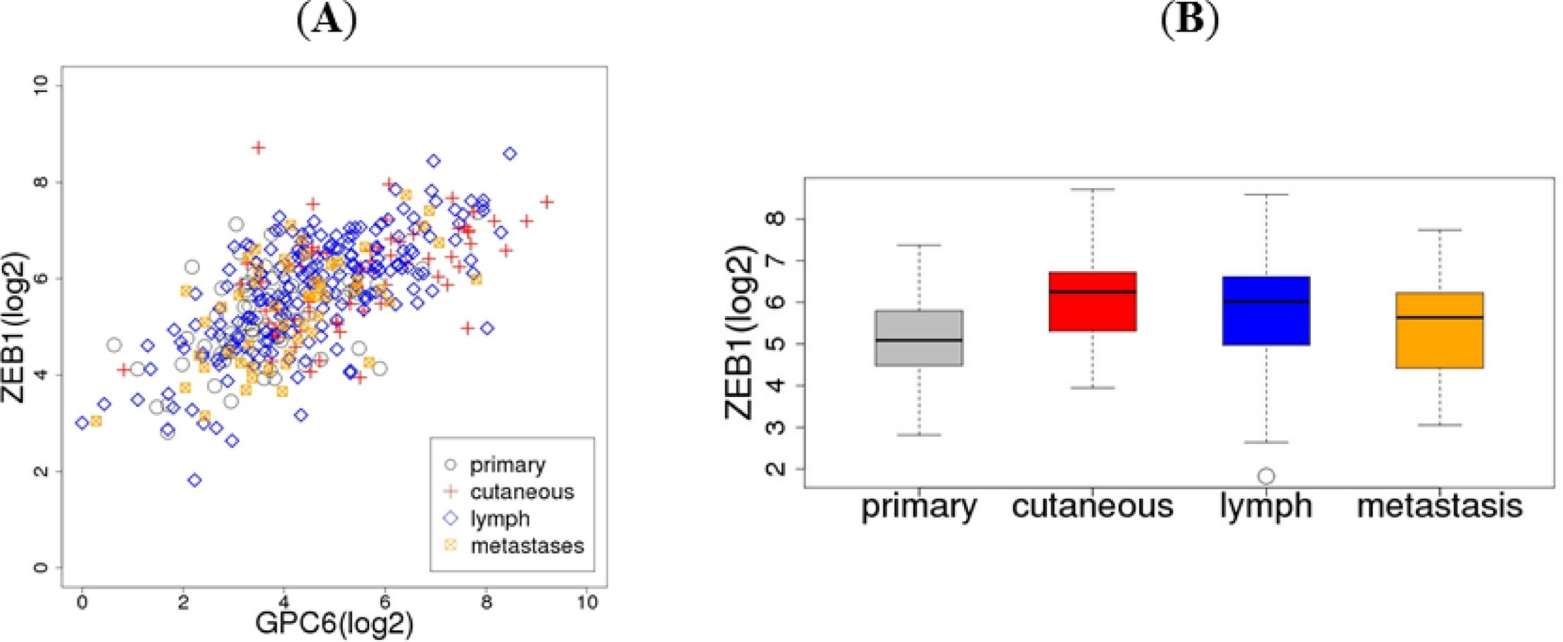
Correlation between GPC6 and ZEB1 expression. (A) GPC6 expression is correlated with ZEB1 in TCGA cutaneous skin melanoma samples. (B) Boxplots of RNA-seq expression levels of ZEB1 in TCGA cutaneous melanoma samples from primary site (skin), and metastases to cutaneous/subcutaneous tissue, lymph node, and distant sites.

ZEB1 exhibited the highest correlation with that of GPC6 (r = 0.63, Spearman correlation) when all 389 melanoma samples were combined. It was also among the ten most positively correlated genes when samples from each of the four categories were analyzed separately (Table 3 and Fig 4A). ZEB1, a zinc finger transcription factor, is an important regulator of the EMT by suppressing the expression of cadherin along with Snail-related zinc-finger transcriptional repressors (SNAIL and SLUG) and other factors such as TWIST [22]. ZEB1 expression was higher in metastatic melanoma (cutaneous/subcutaneous, lymph node, or distant metastases) compared to primary melanoma (Fig 4B). The correlation in expression between GPC6 and ZEB1 is evident within each of the four categories of melanoma. This commonality of correlation within separate categories of melanoma is also true for most of the remaining 82 genes (data not shown).

**Table 3:** Spearman correlation coefficients between GPC6 and the top 10 positively and 5 negatively correlated genes in melanoma RNA-seq samples.

Gene	Melanoma tissue site				Average
	Primary	Cutaneous	Lymph node	Distant metastases	
VCAN	0.57	0.54	0.54	0.68	0.58
SRPX2	0.42	0.70	0.61	0.57	0.58
INHBA	0.51	0.42	0.67	0.71	0.58
TNFAIP6	0.41	0.64	0.65	0.60	0.57
PPAP2B	0.48	0.61	0.55	0.65	0.57
EDIL3	0.43	0.62	0.63	0.56	0.56
PDGFC	0.58	0.49	0.53	0.64	0.56
TCF4	0.50	0.56	0.54	0.63	0.56
COL8A1	0.51	0.48	0.57	0.67	0.56
ZEB1	0.40	0.56	0.65	0.57	0.55
PMEL	-0.36	-0.65	-0.51	-0.47	-0.50
RPUSD3	-0.46	-0.53	-0.46	-0.57	-0.50
UCK1	-0.53	-0.51	-0.43	-0.54	-0.50
TRIM63	-0.35	-0.64	-0.51	-0.57	-0.52
TSPAN10	-0.45	-0.74	-0.49	-0.50	-0.54

We also computed the pair-wise correlation in gene expression between GPC6 and all other genes in each of the 32 TCGA tumor types (9,511 samples) (S1 Table). The overall rank of correlation between each gene and GPC6 across all 32 TCGA tumors was computed using a robust ranking method [23]. The top 200 genes with the highest positive correlation are listed in S2 Table. Fifty (S3 Table) of the top 200 genes are highly significantly associated with cell adhesion (p = 1.7E-22) (Table 4). Genes associated with angiogenesis (THY1, CXCL12, COL15A1, CTGF, ENDRA, ENPEP, MMP14, NRP1, ROBO1, SLIT2, and THBS1) and protein kinase signaling (HTR2A, F2R, EDNRA, HGF, ITGA1, LRP1, PDGFC, THBS1, TGFB3, and ZEB2) were also significantly enriched (p = 3.3E-04 and 2.3E-02, respectively). Key EMT transcription factors were ranked among the top 500 (ZEB1 ranked 88^th^; ZEB2, 148^th^; SNAI1, 422^nd^; SNAI2, 224^th^; and TWIST1, 385^th^).

**Table 4:** Significant GO terms (GOTERM_BP_ALL) that are associated with the top 200 genes mostly correlated with GPC6 across all 33 TCGA tumors.

Biological process	Number of genes in GO term	Multiple-testing-adjusted p-value
Cell adhesion	50	1.7E-22
Biological adhesion	50	9.0E-23
Extracellular matrix organization	22	5.1E-18
Extracellular structure organization	24	2.5E-16
Developmental process	86	1.1E-14
Multicellular organismal development	79	4.7E-13
Blood vessel development	24	1.4E-12
Vasculature development	24	2.1E-12
Anatomical structure development	72	2.9E-12
System development	67	2.7E-11

Similarly, we computed the pair-wise Spearman correlation between the expression levels of GPC6 and those of all other genes in the 1,156 CCLE RNA-seq samples [24]. GO analysis [20] suggested that the top 200 most positively correlated genes were enriched with terms such as development and cell adhesion (S4 Table). Forty-five of the top 200 genes were also among the top 200 identified from the TCGA pan-cancer tumor samples.

## Discussion

Tumor metastasis is the main driver of cancer-related death. It is a complex process that is thought to involve several steps including EMT, invasion, and angiogenesis [25–27]. Many key signaling pathways have been implicated in EMT [22] including those associated with receptor tyrosine kinase [28], the transforming growth factor β (TGFB) superfamily [29], Wnt [30], NOTCH [31], and Hedgehog [32].

GPC6 was up-regulated in a melanoma cell line compared to normal melanocytes. Its expression was also up-regulated in melanoma that had metastasized to cutaneous/subcutaneous tissues, lymph nodes or distant metastases compared to expression in primary melanoma. In melanomas, genes coordinately expressed with GPC6 were largely those involved in cell adhesion and migration including INHBA, PDGFC, PDGFRA, PPAP2B, SPRX2, TCF4, and TNFAIP6 and ZEB1. Pan-cancer analysis using ~9,500 RNA-seq samples from 32 different tumors also confirmed that genes with the highest correlation in expression levels with GPC6 across the 32 tumors were enriched with genes related to cell adhesion and migration. The correlation between GPC6 expression and the expression of key EMT transcription factors (ZEB1/2, TWIST1, and SNAI1/2) among the top 500 (out of ~20,000 genes) suggests that GPC6 may be associated with EMT.

Glypicans are thought to regulate the signaling of Wnt, Hedgehogs, fibroblast growth factors (FGFs) and bone morphogenetic proteins (BMPs) [6–12]. The most studied glypican is GPC3. GPC3 promotes the growth of hepatocellular carcinoma through the Wnt pathway [33, 34] and the activated extracellular signal-regulated kinase (ERK) pathway [35]. GPC3 protein expression is elevated in several neoplastic tissues including melanoma compared to nonneoplastic and normal tissues [36]. Contrarily, down-regulation of GPC3 in ovarian cancer [37] and breast cancer [38] promotes tumor migration and invasion. GPC1, another member of the glypican family, is overexpressed in human breast cancer, and its overexpression may promote tumorigenesis [39]. Although little is known about the role of GPC6 in tumor progression and metastasis in melanoma and other tumors, one would speculate that GPC6 functions also through Wnt, FGF, Hedgehog, and BMP signaling.

To determine a potential mechanism of GPC6 regulation during cancer progression, we hypothesized that GPC6 was targeted by miR-509-3p. It has previously been shown that miR-509-3p was downregulated in gastric cancer, while it was upregulated in gastrointestinal stromal tumors of epithelioid and mixed histological types compared to spindle type [40], metastatic melanoma compared to skin samples from normal healthy donors [41], and in metastatic (CRL-1676 and Sk-mel-28) cells compared to primary (CRL-1675) melanoma cells [42]. It has also previously been shown that miR-509-3p functions in cell growth, proliferation, and migration in gastric cancer [43], renal cell carcinoma [44], and ovarian cancer [21].

## Conclusions

GPC6 expression was elevated in melanoma samples compared to normal melanocytes and elevated in melanomas that had metastasized to regional cutaneous/subcutaneous tissue, lymph node, or distant organs compared to primary melanomas. GPC6 expression was positively correlated with expression of many genes that are involved in cell adhesion and migration in melanoma samples as well as in samples from other tumors from TCGA. We showed that overexpression of miR-509-3p mimic in A375 melanoma cells suppressed GPC6 expression. It has previously been shown that miR-509-3p mimics also suppressed expression of GPC6 and other transcripts (e.g., SNAI2 and TWIST) associated with EMT in ovarian cancer cell lines [21]. Taken together, these results suggest that GPC6 may play a role in and serve as a biomarker for tumor metastatic progression and GPC6 expression may be regulated by miR-509-3p.

## Supporting information

S1 Fig Boxplots of the expression of the ten most frequently selected genes. Both RNA-seq and clinical classification data were obtained from TCGA.
(TIF)

S2 Fig Boxplots of the expression of miR-205 in melanoma samples. Both RNA-seq and clinical classification data were obtained from TCGA.
(TIF)

S1 Table TCGA tumor samples analyzed by RNA-seq (May 2015). (DOCX)

S2 Table Top 200 most correlated genes with GPC6. Each cell lists the Pearson correlation coefficient between the expression level of GPC6 and that of the gene (row) in a TCGA tumor type (column).
(XLSX)

S3 Table The 50 cell adhesion genes among the top 200 most highly correlated with GPC6 across the 32 TCGA tumors. (DOCX)

S4 Table Top 10 significant GO terms (GOTERM_BP_ALL) that are associated with the top 200 genes mostly correlated with GPC6 in 1,156 CCLE samples. (DOCX)
