# Supplementary figures and images for: Glypican 6 is a putative biomarker for metastatic progression of cutaneous melanoma
Source: PLoS One. 2019 Jun 14;14(6):e0218067. doi: 10.1371/journal.pone.0218067 (PMC6568403; doi:10.1371/journal.pone.0218067)

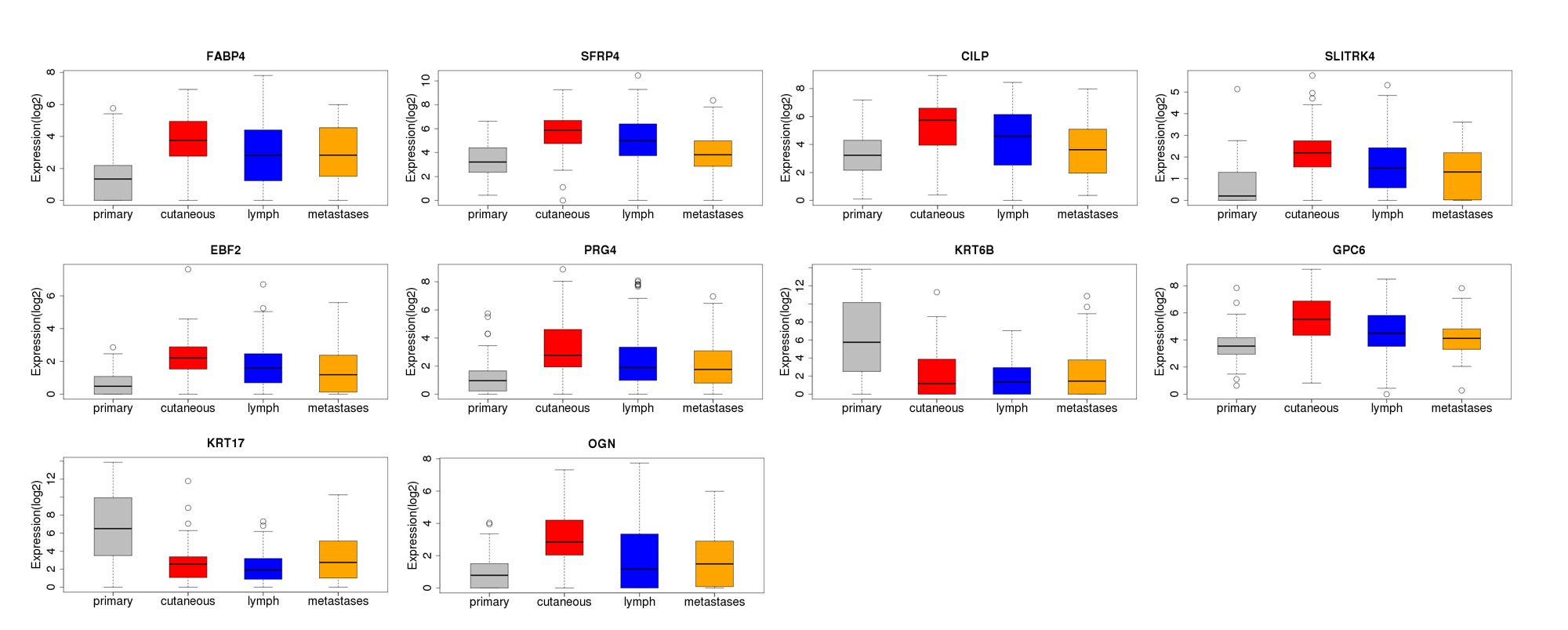

Supplement: S1 Fig — Both RNA-seq and clinical classification data were obtained from TCGA. (TIF) [file pone.0218067.s001.tif]

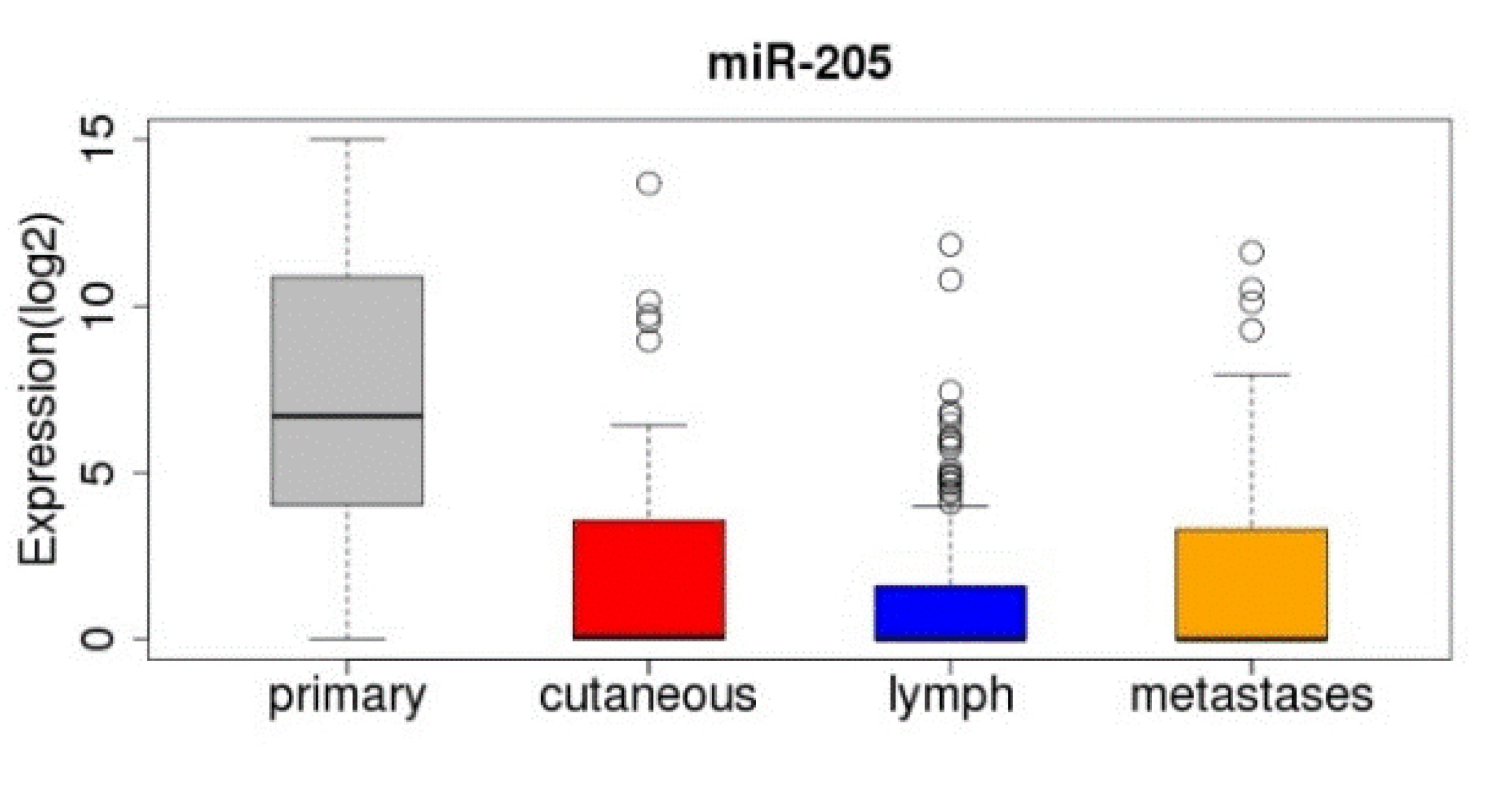

Supplement: S2 Fig — Both RNA-seq and clinical classification data were obtained from TCGA. (TIF) [file pone.0218067.s002.tif]
