# Supplementary material for: Glypican 6 is a putative biomarker for metastatic progression of cutaneous melanoma
Source: PLoS One. 2019 Jun 14;14(6):e0218067. doi: 10.1371/journal.pone.0218067 (PMC6568403; doi:10.1371/journal.pone.0218067)
Supplement: S1 Table — (DOCX) [file pone.0218067.s003.docx]

**S1 Table. TCGA tumor samples analyzed by RNA-seq (May 2015)**

| TCGA Tumor | Description | No. of RNA-seq samples |
| --- | --- | --- |
| ACC | Adrenocortical carcinoma | 79 |
| BLCA | Bladder Urothelial Carcinoma | 408 |
| BRCA | Breast invasive carcinoma | 1102 |
| CESC | Cervical squamous cell carcinoma and endocervical adenocarcinoma | 306 |
| CHOL | Cholangiocarcinoma | 36 |
| COAD | Colon adenocarcinoma | 287 |
| DLBC | Lymphoid Neoplasm Diffuse Large B-cell Lymphoma | 48 |
| GBM | Glioblastoma multiforme | 169 |
| HNSC | Head and Neck squamous cell carcinoma | 522 |
| KICH | Kidney Chromophobe | 66 |
| KIRC | Kidney renal clear cell carcinoma | 534 |
| KIRP | Kidney renal papillary cell carcinoma | 291 |
| LAML | Acute Myeloid Leukemia | 173 |
| LGG | Brain Lower Grade Glioma | 534 |
| LIHC | Liver hepatocellular carcinoma | 374 |
| LUAD | Lung adenocarcinoma | 517 |
| LUSC | Lung squamous cell carcinoma | 502 |
| MESO | Mesothelioma | 87 |
| OV | Ovarian serous cystadenocarcinoma | 266 |
| PAAD | Pancreatic adenocarcinoma | 179 |
| PCPG | Pheochromocytoma and Paraganglioma | 184 |
| PRAD | Prostate adenocarcinoma | 498 |
| READ | Rectum adenocarcinoma | 95 |
| SARC | Sarcoma | 263 |
| SKCM | Skin Cutaneous Melanoma | 473 |
| STAD | Stomach adenocarcinoma | 415 |
| TGCT | Testicular Germ Cell Tumors | 156 |
| THCA | Thyroid carcinoma | 513 |
| THYM | Thymoma | 120 |
| UCEC | Uterine Corpus Endometrial Carcinoma | 120 |
| UCS | Uterine Carcinosarcoma | 57 |
| UVM | Uveal Melanoma | 80 |
