# Supplementary material for: Glypican 6 is a putative biomarker for metastatic progression of cutaneous melanoma
Source: PLoS One. 2019 Jun 14;14(6):e0218067. doi: 10.1371/journal.pone.0218067 (PMC6568403; doi:10.1371/journal.pone.0218067)
Supplement: S3 Table — (DOCX) [file pone.0218067.s005.docx]

**S3 Table.** The 50 cell adhesion genes among the top 200 most highly correlated with *GPC6* across the 32 TCGA tumors

| Gene | Description |
| --- | --- |
| ADAM12 | ADAM metallopeptidase domain 12 |
| AEBP1 | AE binding protein 1 |
| CD93 | CD93 molecule |
| EDIL3 | EGF-like repeats and discoidin I-like domains 3 |
| FAT3 | FAT tumor suppressor homolog 3 (Drosophila) |
| FAT4 | FAT tumor suppressor homolog 4 (Drosophila) |
| THY1 | Thy-1 cell surface antigen |
| WISP1 | WNT1 inducible signaling pathway protein 1 |
| ANTXR1 | anthrax toxin receptor 1 |
| CDH11 | cadherin 11, type 2, OB-cadherin (osteoblast) |
| CDH2 | cadherin 2, type 1, N-cadherin (neuronal) |
| CXCL12 | chemokine (C-X-C motif) ligand 12 (stromal cell-derived factor 1) |
| COL3A1 | collagen, type III, alpha 1 |
| COL5A1 | collagen, type V, alpha 1 |
| COL6A1 | collagen, type VI, alpha 1 |
| COL6A2 | collagen, type VI, alpha 2 |
| COL6A3 | collagen, type VI, alpha 3 |
| COL8A1 | collagen, type VIII, alpha 1 |
| COL11A1 | collagen, type XI, alpha 1 |
| COL12A1 | collagen, type XII, alpha 1 |
| COL15A1 | collagen, type XV, alpha 1 |
| CTGF | connective tissue growth factor |
| DDR2 | discoidin domain receptor tyrosine kinase 2 |
| EMILIN1 | elastin microfibril interfacer 1 |
| ECM2 | extracellular matrix protein 2, female organ and adipocyte specific |
| FERMT2 | fermitin family homolog 2 (Drosophila) |
| FN1 | fibronectin 1 |
| FLRT2 | fibronectin leucine rich transmembrane protein 2 |
| HSPG2 | heparan sulfate proteoglycan 2 |
| ISLR | immunoglobulin superfamily containing leucine-rich repeat |
| ITGA1 | integrin, alpha 1 |
| ITGA11 | integrin, alpha 11 |
| ITGA5 | integrin, alpha 5 (fibronectin receptor, alpha polypeptide) |
| ITGAV | integrin, alpha V (vitronectin receptor, alpha polypeptide, antigen CD51) |
| ITGBL1 | integrin, beta-like 1 (with EGF-like repeat domains) |
| LAMA2 | laminin, alpha 2 |
| LAMA4 | laminin, alpha 4 |
| LAMB1 | laminin, beta 1 |
| NRP1 | neuropilin 1 |
| NTM | neurotrimin |
| NID1 | nidogen 1 |
| NID2 | nidogen 2 (osteonidogen) |
| OMD | osteomodulin |
| POSTN | periostin, osteoblast specific factor |
| PCDH18 | protocadherin 18 |
| ROBO1 | roundabout, axon guidance receptor, homolog 1 (Drosophila) |
| SRPX | sushi-repeat-containing protein, X-linked |
| THBS1 | thrombospondin 1 |
| THBS2 | thrombospondin 2 |
| VCAN | versican |
