# Supplementary material for: Glypican 6 is a putative biomarker for metastatic progression of cutaneous melanoma
Source: PLoS One. 2019 Jun 14;14(6):e0218067. doi: 10.1371/journal.pone.0218067 (PMC6568403; doi:10.1371/journal.pone.0218067)
Supplement: S4 Table — (DOCX) [file pone.0218067.s006.docx]

**S4 Table.** Top 10 significant GO terms (GOTERM_BP_ALL) that are associated with the top 200 genes mostly correlated with GPC6 in 1,156 CCLE samples

| GO Term | Number of genes in the GO term | Multiple testing adjusted p-value |
| --- | --- | --- |
| system development | 84 | 6.6E-9 |
| multicellular organism development | 91 | 7.8E-9 |
| single-organism developmental process | 97 | 7.8E-9 |
| anatomical structure development | 98 | 1.0E-8 |
| developmental process | 98 | 1.3E-8 |
| cell adhesion | 46 | 1.7E-7 |
| biological adhesion | 46 | 1.6E-7 |
| extracellular structure organization | 20 | 2.6E-7 |
| extracellular matrix organization | 20 | 2.8E-7 |
| single-multicellular organism process | 97 | 5.0E-7 |
